# Estimation of new HIV diagnosis rates among high‐risk, PrEP‐eligible individuals using HIV surveillance data at the Metropolitan Statistical Area level in the United States

**DOI:** 10.1002/jia2.25433

**Published:** 2019-12-20

**Authors:** Robertino Mera, Susan Scheer, Christoph Carter, Moupali Das, Julius Asubonteng, Scott McCallister, Jared Baeten

**Affiliations:** ^1^ Gilead Sciences, Inc Foster City CA USA; ^2^ San Francisco Department of Public Health San Francisco CA USA; ^3^ Departments of Global Health, Medicine and Epidemiology University of Washington Seattle WA USA

**Keywords:** modelling, PrEP, HIV prevention, public health, HIV epidemiology, HIV prevention trials

## Abstract

**Introduction:**

New HIV diagnoses have fallen in the past decade due to increased HIV testing, earlier diagnosis, earlier antiretroviral treatment, improved linkage to care and engagement in care, and the recent increased uptake of pre‐exposure prophylaxis (PrEP). We propose a novel method to compute the rate of new HIV diagnoses at the Metropolitan Statistical Area (MSA) level in the US to support the evaluation of comprehensive treatment and prevention efforts over time.

**Methods:**

The number of new HIV diagnoses, number of individuals with a PrEP indication and aggregated person‐time exposed to PrEP during the years 2012 to 2017 were used to compute a new HIV diagnosis rate for people at risk of HIV excluding those already on PrEP for the 105 MSAs in the US with published HIV surveillance data. In our calculation of person‐time with a PrEP indication, time‐at‐risk excluded time on PrEP and time after an HIV diagnosis. We used a multivariate Poisson regression model to estimate HIV diagnosis rates by year and location.

**Results:**

From 2012 to 2017, the aggregate HIV diagnoses rate among high‐risk individuals with an indication for PrEP in the 105 MSAs decreased from 4.14 per 100 person‐years (PY) (95% CI 4.10 to 4.19) to 3.26 per 100 PY (95% CI 3.22 to 3.30). For the 25 US MSAs that overlapped with an ongoing large randomized clinical trial of PrEP in men who have sex with men (MSM), the HIV diagnosis rate from 2012 to 2017 decreased from 4.86 per 100 PY (95% CI 4.80 to 4.93) to 3.61 per 100 PY (95% CI 3.56 to 3.66), a decline that was more rapid than in non‐study MSAs (IRR for trial site 1.19, 95% CI 1.18 to 1.20).

**Conclusions:**

We propose a model to estimate the background HIV diagnosis rate in people at risk for HIV and with a PrEP indication in US MSAs (excluding those on PrEP) using publically available surveillance data which can evaluate trends over time. Data generated using this methodology could be used by policy makers and local HIV prevention specialists to evaluate and monitor their prevention efforts for the population at risk in their communities.

## 1 Introduction

New HIV diagnoses have fallen over the past decade due to the following: (1) increased HIV testing, (2) earlier diagnosis of HIV infection, (3) earlier antiretroviral treatment for people living with HIV (treatment as prevention (TasP)), (4) improved linkage to care and engagement in care, and, (5) the recent increased uptake of pre‐exposure prophylaxis (PrEP), the use of antiretroviral medications in people at risk of HIV to reduce HIV acquisition 1. Tenofovir disoproxil fumarate plus emtricitabine (F/TDF) was approved for PrEP in 2012 after the demonstration that the prophylactic use of F/TDF could substantially reduce the risk of HIV acquisition in high‐risk populations 2, 3. Subsequently the CDC, WHO and IAS‐USA have issued guidance recommending PrEP as part of HIV prevention strategies in high‐risk populations 4, 5, 6. Immediately post‐approval the use of F/TDF for PrEP was low, with fewer than 10,000 individuals initiating PrEP per year in 2012 and 2013 (Gilead Sciences Inc. internal data). In 2014 PrEP use began to rise rapidly, with over 100,000 new PrEP starts in 2018 and an estimated 202,000 current PrEP users in the US (Gilead Sciences Inc. internal data) 7.

In many urban centres, the uptake of PrEP has correlated with a decrease in the number of new HIV diagnoses 8, 9, 10, 11. Furthermore, a recent analysis demonstrated that the US states with the highest PrEP use had larger reductions in HIV diagnosis rates, an impact that was independent of TasP 12, 13. However, while PrEP uptake has increased dramatically, it is only being utilized by approximately 18% of the estimated 1.2 million people living in the US who have an indication for PrEP 14. The heterogeneity in the HIV epidemic as well as in the implementation of comprehensive prevention, treatment, and care efforts such as TasP and PrEP have led to significant geographic variance in HIV diagnosis rates, ranging from 1.5/100 PY to 35.3/100 person‐years (PY) in major metropolitan statistical areas (MSA) 1.

The diversity in the HIV epidemic and the heterogeneity in HIV diagnoses rates across communities in the US make it challenging to estimate the population‐level impact of PrEP in reducing new HIV diagnoses in real‐word settings. The challenge stems partially from the need to understand the rate of new HIV infections specifically in the subgroup of high‐risk individuals with an indication for PrEP (here referred to as PrEP‐eligible individuals). Since new HIV infection diagnosis rates vary substantially by community, per‐community or aggregate estimates of PrEP effectiveness require knowledge of new diagnosis rates within specific communities. Such community‐level data are important to guide local prevention efforts tailored to each community's specific needs. It is equally important to understand local HIV diagnosis rates in PrEP‐eligible individuals when designing and interpreting clinical trials involving PrEP and novel HIV prevention strategies, especially when placebo arms must be omitted for ethical reasons 15. As F/TDF efficacy is high when taken as directed, the lack of a placebo or control arm can make it challenging to determine whether a new drug for PrEP is effective 16.

Here we present a computational approach that utilizes publicly available MSA‐level HIV surveillance data collected annually by the CDC in order to calculate the rate of new HIV diagnosis among PrEP‐eligible individuals. By doing this, we sought to quantify differences in new HIV diagnosis rates by community in PrEP‐eligible individuals, and to establish a background diagnosis rate, analogous to a placebo rate. Using this approach, we have generated an estimate of the rate of new HIV diagnoses among PrEP‐eligible individuals on an MSA level. As an example of this method's utility, we demonstrate how this rate varies over time in MSAs participating in an ongoing PrEP clinical trial compared to control MSAs that are not participating in the trial. This methodology for estimating a background rate of new HIV diagnoses among PrEP‐eligible individuals could be useful for public health and policy officials assessing the impact of HIV prevention efforts on a community scale, and for creating a hypothetical placebo arm in active comparator PrEP clinical trials.

## 2 Methods

### 2.1 Data sources

Data were obtained from publically available sources with the exception of patient‐level PrEP usage data, which was obtained from a pharmacy claims database which provided continuous or discontinuous TVD exposure periods (Symphony Health, Phoenix, AZ, USA). The number of new HIV diagnoses in 136 MSAs from 2012 to 2017 were obtained from CDC HIV Surveillance Reports 1. The estimated number of adults in specific HIV transmission groups with indications for PrEP nationally and by jurisdiction in 2015 were obtained from published CDC tables 14. Data on state population totals and components of change from 2010 to 2017 were obtained from US Census data 17. Data on metropolitan population totals were obtained from US census data 18. PrEP utilization data at the MSA level were derived using a validated algorithm which removes from the calculation HIV treatment and off label chronic hepatitis B use 19. PrEP prevalence data by state and three digit zip code from 2012 to 2017 were obtained from AIDSVu 20.

### 2.2 Statistical/epidemiological analyses

The proportion of adults with a PrEP indication by MSA is computed using the published data by state and US Census data. The temporal changes by calendar year are computed using the natural rate of change in each MSA overall population as per the census estimates.

The rate of new HIV diagnoses per 100 PY is obtained as follows: The numerator corresponds to the count of new adult HIV diagnoses by MSA on a given year as reported by CDC, which is adjusted so that those without a PrEP indication are removed from the calculation by counting only those individuals with a transmission category (MSM, PWID, HET) who are included in the denominator. These excluded individuals (non‐PrEP risk categories) represented 0.19% of all new diagnoses. The denominator corresponds to person‐time for individuals who are (1) HIV seronegative, (2) have an indication for PrEP, and (3) are not taking PrEP. The denominator is calculated using a survival analysis interval censoring approach, by starting with the number of adults with a PrEP indication alive at the beginning of the period multiplied by one year of follow up, minus the number of adult PrEP users prevalent during the same period multiplied by the average person time not at risk, minus the number of adult new HIV cases multiplied by the average person time at risk, assuming that the count of cases follows a Poisson distribution and that the time between events follows an exponential distribution. This approach censors the individuals at risk in the denominator who acquired HIV at the midpoint of the interval, with those dying during the interval being also censored, and both not contributing time in the next interval. Additionally, this approach includes in the denominator only person‐time that individuals were at risk and not taking PrEP during a given year excluding the small number who become HIV positive during the period 21.

More specifically, let

N = number of HIV diagnoses
n = Number of HIV diagnoses without PrEP indications
PT = Total person time for those with PrEP indications
PT_PrEP_ = Total person time of subjects on PrEP during exposure to TVD
PT_HIV_ = Total person time of subjects who became HIV positive

$$\text{Incidence}\;\text{rate}=\frac{\left(N-n\right)}{\text{PT}-{\text{PT}}_{\text{PrEP}}-{\text{PT}}_{\text{HIV}}}$$

Moreover PT = number of subjects with a PrEP indication alive at the start of the observation period times one year. PT_PrEP_ = cumulative periods of time exposed to TVD minus the time after and if they become HIV positive (1 per 100 person years of TVD exposure). PT_HIV_ = amount of time during the observation period after subjects become HIV positive during the interval.

We computed an aggregate rate of new HIV diagnoses among PrEP‐eligible individuals by performing the aforementioned calculation using merged data from 105 MSAs that had complete PrEP indication information available. We also performed identical calculations with MSAs stratified by overlap with the DISCOVER trial, an ongoing double blind active controlled study of F/TDF versus emtricitabine plus tenofovir alafenamide (F/TAF) for PrEP being conducted in 2,500 men who have sex with men (MSM) and transwomen 22. These calculations were used in a multivariate Poisson regression model estimating yearly HIV diagnosis incidence rate among individuals with a PrEP indication over 2012 to 2017. The offset of the model is the denominator above (person time at risk) and the covariates are time and a binary variable (0/1) which is 1 if the MSA in question contains a DISCOVER site and 0 otherwise. 95% Confidence intervals were computed using exact Poisson methods.

We performed a sensitivity analysis using self‐reported testing information at the MSA level and explored the potential bias due to differential proportions of HIV testing over time and place. In addition, we conducted a sensitivity analysis using precise MSM HIV incidence rate estimates from San Francisco (which has a robust HIV diagnosis and reporting programme). Statistical analyses were carried out using STATA 15.

### 2.3 Ethics statement

All data used in this manuscript were obtained from publically available databases and publications with the exception of some patient‐level PrEP utilization data which were obtained in an anonymized form from a commercial source (Symphony Health).

## 3 Results

**Figure 1:**
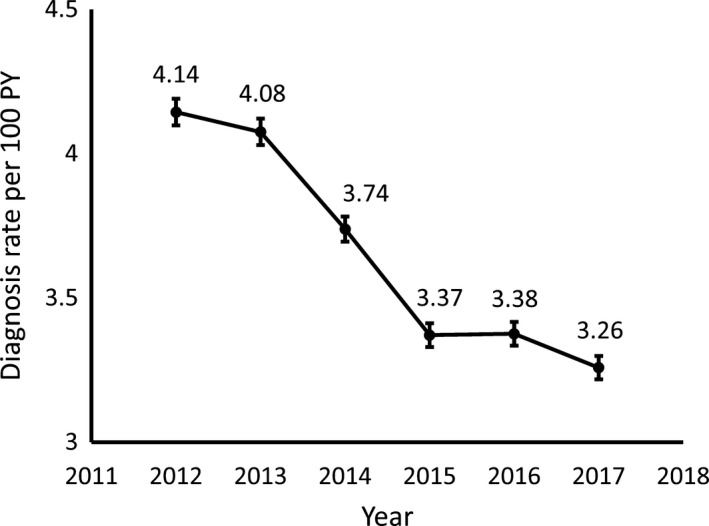
Estimated HIV diagnosis rate in PrEP‐eligible individuals. A Poisson regression model incorporating state and MSA‐level data on population, HIV diagnosis rate and HIV risk factors was used to generate an estimate of HIV diagnosis rates across 105 MSAs as described in the Section 2. Error bars represent 95% confidence intervals from the multivariate model.

Using merged data from 105 MSAs that had complete PrEP indication information available, we calculated the estimated rate of new HIV diagnoses among PrEP‐eligible individuals between 2012 and 2017. Over that period, we observed a decrease in the rate of new HIV diagnoses, from 4.14 per 100 PY (95% CI 4.10 to 4.19) to 3.25 per 100 PY (95% CI 3.22 to 3.30), a mean decrease of 6.0% per year (95% CI 5.3 to 6.4) (Figure 1). The largest drop occurred between 2013 and 2015, with a more gradual decline occurring from 2015 to 2017. A similar method approximating HIV diagnosis rates solely among MSM revealed rates consistent with those observed in the overall PrEP‐eligible population (data not shown).

**Figure 2:**
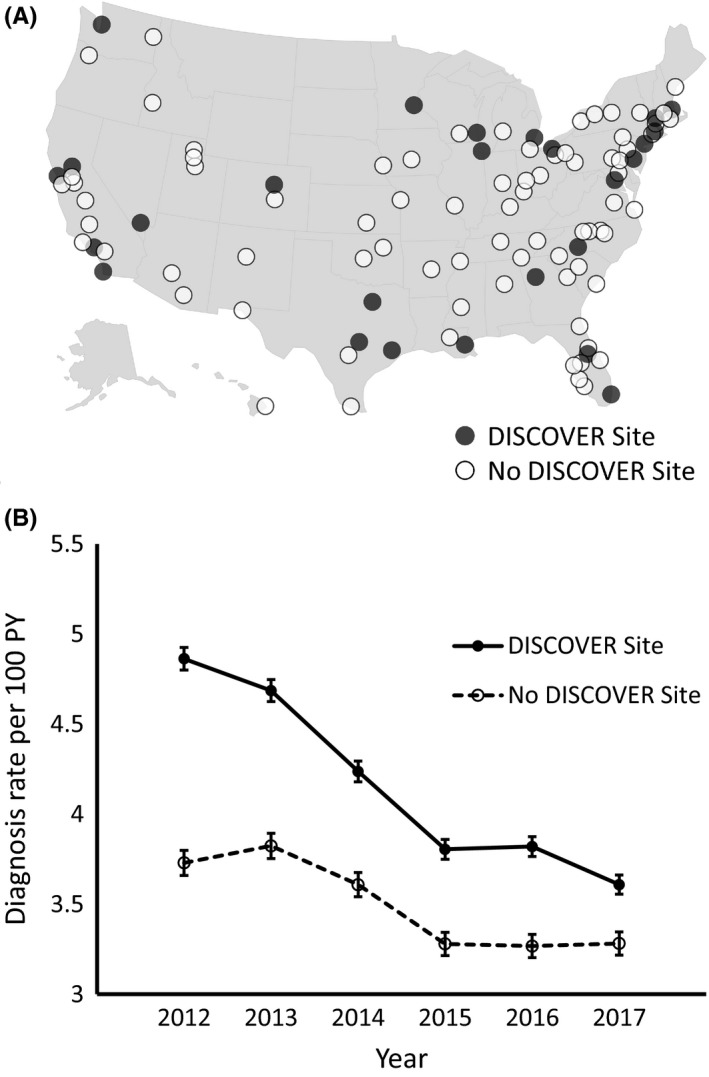
Estimated HIV diagnosis rate in PrEP‐eligible individuals in MSAs participating in an ongoing PrEP clinical trial. **(A)** Map depicting MSAs included in the calculations. MSAs with a DISCOVER site are in black, those without are in white. **(B)** A Poisson regression model incorporating state and MSA‐level data on population, HIV diagnosis rate, and HIV risk factors was used to generate an estimate of HIV diagnosis rates across 105 MSAs, stratified by the presence of a PrEP clinical trial (DISCOVER) site, as described in the Methods section.

To demonstrate the utility of this method, we compared HIV diagnosis rates in MSAs stratified by overlap in the DISCOVER trial, an ongoing large randomized active‐controlled PrEP trial 22. Twenty‐five of the 105 MSAs contained PrEP clinical trial sites (23.8%), whereas 80 (76.2%) did not (Figure 2A). We used data from these sites to inform a DISCOVER site versus time interaction model in order to compare the change in diagnosis rates over time. As demonstrated in Figure 2B, the MSAs with DISCOVER trial clinical sites had a significantly higher rate of new HIV diagnoses compared to those that did not contain trial sites throughout the period 2012 to 2017. The estimated rate of HIV diagnoses declined rapidly in MSAs with DISCOVER sites, from 4.86 (95% CI 4.80 to 4.92) in 2012 to 3.61 (95% CI 3.56 to 3.66). The estimated rate of new HIV diagnoses also declined in non‐DISCOVER MSAs, albeit at a lower rate, from 3.73 (95% CI 3.66 to 3.80) in 2012 to 3.28 (95% CI 3.22 to 3.35) in 2017. Of note, the DISCOVER site MSAs saw a decline in new HIV diagnoses between 2016 and 2017 (after initiation of the DISCOVER trial), whereas the rates in the non‐DISCOVER MSAs remained stable. The overall reduction in diagnosis rates in DISCOVER MSAs was faster than in non‐DISCOVER site MSAs (IRR for trial site 1.19, 95% CI 1.18 to 1.20).

We conducted sensitivity analyses to assess this method of estimating new HIV diagnosis rate among high‐risk populations. To assess the possibility that the observed changes in HIV diagnosis rates could be due to changes in the HIV testing practices (rather than changes in the rate of new HIV infections), we utilized MSA‐level HIV testing data from the CDC Behavioural Risk Factor Surveillance System 23 to evaluate the impact of differential testing by time and MSA. This analysis revealed a 12.7% underestimation of the incidence rate, which was not significantly dependent on time or MSA. We also conducted a sensitivity analysis using data from the San Francisco Department of Public Health (SFDPH), which has enhanced local HIV epidemiology data such as MSM population size and PrEP usage. Using these estimates from the SFDPH 24 in our model, we calculated a rate of new HIV diagnoses of 2.9 per 100 PY (95% CI 2.6 to 3.2) in 2013 among MSM with a PrEP indication. Additionally, we found that our estimates were comparable to the incidence rates reported from a prospective cohort of initially seronegative persons in New York City with 34,455 PY of follow‐up between 2009 and 2012 (5.7 per 100 PY for men, 95% CI 5.2 to 6.3) 25.

## 4 Discussion

The methodology we present here uses publically available HIV surveillance data to calculate estimated HIV diagnosis rates among PrEP‐eligible individuals while excluding those on PrEP. Having the background rate of HIV diagnosis in those who are not on PrEP allows for a comparison between the observed rate of new HIV diagnoses post intervention with the expected rate over time and may be useful for evaluating the impact of HIV prevention efforts, such as the proposed federal government effort to improve PrEP coverage in certain counties and states. Furthermore, computation of an incidence rate for certain risk factors is possible using the same data sources. The overall rate in 2017 among MSM who represent (69.7%) of all cases was 3.42 per 100 PY, 95% CI 3.37 to 3.47. Using the same method and the availability of race both for numerator and denominator, 2017 rates can be computed for Whites (2.94 per 100 PY, 95% CI 2.87 to 3.01), Hispanics (3.67 per 100 PY, 95% CI 4.22 to 4.43) and African Americans (3.45 per 100 PY, 95% CI 3.38 to 3.51).

In addition, we used this methodology to calculate an estimated background rate of HIV diagnosis, analogous to a placebo rate, in the MSAs where a large PrEP trial (DISCOVER) is being conducted. The methodology revealed several notable findings. First, the HIV diagnosis rates in the DISCOVER MSAs was higher overall than in the non‐DISCOVER MSAs, likely reflecting the efforts to conduct the trial in geographies with high historic HIV prevalence and current higher incidence. Second, we demonstrate that the temporal declines in new HIV diagnosis rates differed between DISCOVER and non‐DISCOVER MSAs, likely reflecting the aggressive prevention efforts taking place in these higher risk MSAs. And lastly, we observed that the DISCOVER MSAs had a continued decline in the HIV diagnosis rate after the implementation of the DISCOVER trial (2015 to 2017), while rates in non‐MSAs remained steady. While our methodology cannot determine causality and regression toward the mean may be operating, the findings demonstrate how our methodology could be useful in tracking the impact of PrEP and other prevention strategies on an MSA level over time.

This approach may also be useful in the design and interpretation of active comparator noninferiority trials for HIV prevention. Since a true placebo arm is often excluded in modern PrEP clinical trials 15, trial design and interpretation rely on historical placebo and prior active comparator efficacy data. This approach assumes that the active comparator is similarly effective compared to prior clinical trials, and thus the interpretation of the trial may be incorrect if the active comparator performs significantly better (or worse) than it has previously. This is of particular concern in modern PrEP trials where efficacy has improved due to increased adherence to PrEP as compared to first generation of PrEP trials. Furthermore, the background rate of HIV infection may be different depending on geographical area and may have changed significantly over time due to both PrEP as well as TasP and other prevention efforts. In this case, inclusion of an estimated background HIV diagnosis rate can function as a computational “placebo” rate of infection, helping in determining an appropriate non‐inferiority (NI) margin, and in the appropriate interpretation of NI trial data.

We note several limitations in the approach reported here. Because CD4 count data stratified by PrEP indication are not available, it was not possible to use the well‐characterized CD4 depletion model to estimate incidence rates 26, and thus we used new diagnosis rate as a surrogate for incidence. This approach could potentially introduce bias if HIV testing frequency changes; however, our sensitivity analyses demonstrate that variation in testing frequency does not account for the declines in computed diagnosis rates that we observed. Furthermore, RCTs may not be generalizable to the population at risk. It is possible that the subjects who enroll on a PrEP Clinical Trial may originate in a low transmission group. Another limitation is that MSAs with small populations may produce estimates with a larger variance, and the assumptions may not be appropriate for risk factors.

Additionally, because PrEP indication data are available on a state scale but not an MSA scale, our model relies on the assumption that PrEP indication frequencies within MSAs are equivalent to those in their parent state. While this assumption is imperfect, our MSA PrEP indication estimates are likely underestimates, since the proportion of individuals in the entire state is probably lower than metropolitan areas. Despite these limitations, our findings suggest that a computational HIV diagnosis rate derived using this methodology can provide a reliable estimation of HIV incidence among high‐risk individuals.

Publically available HIV surveillance data can be used to compute an estimated HIV diagnosis rate among high‐risk, PrEP‐eligible individuals. This methodology may be useful for local policy makers and prevention specialists to monitor ongoing prevention efforts. Additionally, data generated using this methodology may be useful for investigators conducting HIV prevention studies, especially when the lack of a placebo arm makes the determination of efficacy challenging.

## 5 Conclusions

The model we propose allows for the estimation of the background HIV diagnosis rate in people at risk for HIV and with a PrEP indication in US MSAs (excluding those on PrEP) using publicly available surveillance data which can evaluate trends over time. Data generated using this methodology could be used by policy makers and local HIV prevention specialists to evaluate and monitor their prevention efforts for the population at risk in their communities.

## Competing interests

RM, JA, CC, MD and SM are employees of Gilead Sciences and hold stock interest in the company.

## Authors' contributions

RM designed the model and computational framework and analysed the data. JA performed the sensitivity analysis. CC wrote the manuscript with input from all authors. JB, MD and SM contributed to study conception, manuscript writing, and were in charge of overall direction and planning. SS provided data for sensitivity analysis and guidance on study design. All authors discussed the results and provided critical review of the manuscript.
